# A Comparative Study of Deterministic and Stochastic Models of Microstructure Evolution during Multi-Step Hot Deformation of Steels

**DOI:** 10.3390/ma16093316

**Published:** 2023-04-23

**Authors:** Piotr Oprocha, Natalia Czyżewska, Konrad Klimczak, Jan Kusiak, Paweł Morkisz, Maciej Pietrzyk, Paweł Potorski, Danuta Szeliga

**Affiliations:** 1 Faculty of Applied Mathematics, AGH University of Science and Technology, al. Mickiewicza 30, 30-059 Krakow, Poland; 2 Faculty of Metals Engineering and Industrial Computer Science, AGH University of Science and Technology, al. Mickiewicza 30, 30-059 Krakow, Polandmpietrz@agh.edu.pl (M.P.); szeliga@agh.edu.pl (D.S.)

**Keywords:** hot metal forming, microstructure evolution, dislocation density, grain size, recrystallization, nucleation, stochastic model

## Abstract

Modern construction materials, including steels, have to combine strength with good formability. In metallic materials, these features are obtained for heterogeneous multiphase microstructures. Design of such microstructures requires advanced numerical models. It has been shown in our earlier works that models based on stochastic internal variables meet this requirement. The focus of the present paper is on deterministic and stochastic approaches to modelling hot deformation of multiphase steels. The main aim was to survey recent advances in describing the evolution of dislocations and grain size accounting for the stochastic character of the recrystallization. To present a path leading to this objective, we reviewed several papers dedicated to the application of internal variables and statistical approaches to modelling recrystallization. Following this, the idea of the model with dislocation density and grain size being the stochastic internal variables is described. Experiments composed of hot compression of cylindrical samples are also included for better presentation of the utility of this approach. Firstly, an empirical data describing the loads as a function of time during compression and data needed to create histograms of the austenite grain size after the tests were collected. Using the measured data, identification and validation of the models were performed. To present possible applications of the model, it was used to produce a simulation imitating industrial hot-forming processes. Finally, calculations of the dislocation density and the grain size distribution were utilized as inputs in simulations of phase transformations during cooling. Distributions of the ferrite volume fraction and the ferrite grain size after cooling recapitulate the paper. This should give readers good overview on the application of collected equations in practice.

## 1. Introduction

Development of the modern industry requires construction materials that combine strength and formability with a high strength-to-density ratio. Crack resistance, creep resistance, wear resistance, and the Charpy impact properties are also significant in some applications. Steels have met these requirements for many decades. Historically, grain refinement was the main strengthening mechanism for steels investigated in the second half of the 20th century, when High Strength Low Alloyed (HSLA) steels were developed [1]. An improvement in strength and workability was obtained by controlling precipitation and its influence on recrystallization [2]. A different strengthening mechanism was used in Advanced High Strength Steels (AHSS), which were developed during 1990s. These steels are composed of soft ferrite with islands of hard constituents of bainite, martensite, and retained austenite. AHSSs benefit from the best features of the phases they are made of and their internal microstructure [3]. These particular composite materials are locally isotropic as they have the same material inside each phase. Nevertheless, they are heterogeneous at the macroscopic scale due to spatial variations of volume fractions of the phases. Modern bainitic steels are another group that benefits from a combination of phases in the microstructure [4,5]. Exceptional properties of metals and alloys with heterogeneous microstructures are discussed in many publications. As far as steels are concerned, recent papers on complex phase [6], multiphase [7], ferritic/martensitic [8], pipeline [9], and bainitic steels [10] should be mentioned. Modern bainitic steels are used for manufacturing rails [11] and rods [12]; the latter mainly has further application as stock for cold heading processes [13].

Dual Phase (DP) and Complex Phase (CP) steels are the two leading examples of the AHSSs with particular applications in car body manufacturing [14,15]. The properties of the DP steels with the microstructure composed of the fine ferrite matrix with dispersed hard islands of martensite have been intensively studied [16]. The high strength and total elongation of DP steels go side-by-side with low local formability caused by large gradients of properties at the phase boundaries. CP steels are characterized by a heterogeneous microstructure with a fine mixture of bainite, martensite, and ferrite. Compared with DP steel, the volume fraction of hard constituents is higher, leading to higher strength. On the other hand, the gradients of properties between microstructural constituents are smaller than those in DP steels. Thus, CP steels with a more heterogeneous microstructure have better local formability [3]. This unique feature makes CP steels more suitable for stretch-forming processes [17]. It was suggested in [18] that more balanced mechanical properties of multiphase steels can be achieved by tailoring the microstructure gradients. 

As far as modern bainitic steels are concerned, extensive research has been conducted during the last decades of the 20th century. Numerous works of Bhadeshia should be mentioned, such as [4,5]. The design of these steels has to be based on understanding the mechanisms of bainite formation. Contrary to the well-documented diffusionless martensitic transformations, modelling of bainite transformation, which involves compositional change, is still a challenge [19]. A significant effect of bainite as a transformation product on mechanical properties is observed, in particular on hardness [20], tensile strength [21], and Charpy impact properties [22], but also on fracture resistance and creep resistance [19]. Bainitic microstructures in steels are obtained by appropriate design of the composition and by proper control of process parameters and manufacturing conditions. Modern bainitic steels contain about 1% of silicon, which allows to form carbide-free bainite with ferrite plates and a high-volume fraction of retained austenite in the form of thin layers or islands [20,23]. Relationships between microstructure and properties of bainitic steels have been intensively studied [19]. The design of bainite in steels with heterogeneous microstructures was also investigated in the Research Fund for Coal and Steel (RFCS) project [24]. Analysis of the Continuous Cooling Transformation (CCT) diagrams for many bainitic steels shows that their microstructures do not consist entirely of bainite. The mechanical properties may vary significantly as a function of the cooling cycle. Experimental research is not sufficient to fully understand the bainite transformation. Appropriate numerical modelling could deliver more valuable findings that should support further experiments. 

Advanced numerical models are needed to predict distributions of microstructural features and design thermal–mechanical cycles that allow us to obtain moderate gradients in the microstructure. The models must be able to predict distributions of various microstructural parameters instead of their average values. Beyond this, since process optimization is the prospective application of these models, they have to be characterized by low computing costs. There are several recent intensive studies for numerical tools which can help in prediction distributions of parameters, leading to realistic modelling of heterogeneous materials. Models considered in the literature usually belong to two main groups models of materials (see PhD thesis [25] for more details and extensive discussion): mean-field and full-field. The full-field models usually describe material more completely and build on various equations for phase transformations, kinetics of the process, microstructure parameters, etc. Examples of models in this group are Integrative Computational Materials and Process Engineering (ICMPE) [26], Digital Material Representation (DMR), and Representative Volume Element (RVE) models [27]. On the other hand, mean-field models are designed relying on averaged parameters of the material microstructure, such as dislocation density or grain size for the sample. Applications of the full-field models to design AHSSs are numerous [28]. Many such papers were published during first decades of the 21st century, though we will not discuss them here. As it has been for the AHSSs, advanced numerical methods are used to design the development of bainitic microstructures for applications in the phase field model [29] or crystal plasticity [30]. The recent solutions simulate the sub-division of austenite grains by bainite sheaves in an explicit way [23]. 

By design, the full-field models provide much better predictive capabilities; however, they require more demanding computations, which in turn result in longer computing times. Thus, the objective is to search for a fast mean-field model with extended predictive capabilities. As shown in [31], the internal stochastic variables can be used to describe the heterogeneous microstructures of alloys, leading to an interesting mean-field model. Such models can then be effectively used for prediction of gradients of microstructural features in designed final products. In the model described in [31], the internal variables account for the history of the process. The stochastic character of these variables enables not only a prediction of average values of parameters but, more importantly, a prediction of their distributions.

The papers, in which the stochastic approach was applied to describe distributions of microstructural features, are usually focused on a correlation between microstructure and in-use products’ properties. Among the recent papers, statistics for geometry description was used in homogenization methods [32] and *n*-point statistics was applied to predict crack probability [33]. In a few papers, the probability distribution of selected parameters was used as an input for a deterministic material model, e.g., the authors of [34] studied the austenite grain size before recrystallization in term of histograms. This study provides clear connections between histograms’ evolution and recrystallization kinetics. The internal variable stochastic model for hot deformation was proposed in our earlier publication [35]. In our model, we introduced a stochastic approach to both dynamic and static recrystallization at elevated temperatures. The stochastic variables were used in equations describing the evolution of the dislocation populations and the grain size. As a consequence, the model may predict distributions of various microstructural parameters. This model was identified based on the experimental data [31]. In publication [36], the model was utilized to conduct simulations of multi-step hot forming processes.

The heterogeneous multiphase microstructures discussed above are obtained during cooling after hot forming. However, the microstructural heterogeneity depends, to a large extent, on the state of the austenite prior to phase transformations. The austenite grain size and dislocation density (when the recrystallization is not completed) strongly influence the kinetics of phase transformations and resulting phase composition. Thus, in our work, we focused on the stochastic modelling of hot forming and prediction of the heterogeneity of the microstructure at the start temperature for transformations (*A_e_*_3_). The task of the hot forming model is to supply the input data (histograms of the dislocation density and the grain size) for the further simulation of the phase transformations.

In summary, there were two objectives of the present work. The first was a review of the stochastic models of recrystallization. Based on this review, we presented a model of nucleation, which is a statistical representation of the stochastic models used in the RVE. As an exemplary practical approach, identification and validation of the stochastic model of a hot deformation of multiphase steels, including dynamic processes during deformation and static processes during interpass times, were performed. The second objective was an exemplary application of this model to the simulation of various technological routes for the hot strip rolling process. Finally, the outputs of these simulations were utilized as an input for modelling phase transformation during various cooling cycles. This example of an application of modelling summarizes the paper’s contents.

## 2. Constitutive Model Based on Internal Variables

Numerous models of materials’ response during hot forming that were developed in the second half of the 20th century only used external variables as arguments in models [37]. In these models, the flow stress is a function of process parameters (e.g., temperature, strain rate), which are grouped in the vector **p**: *σ_p_* = f(**p**). The weak point of these models is that they do not account for the history of the process. Whenever the temperature or the strain rate change rapidly, the calculated flow stress immediately moves to a new equation of state and is a function of the new values of the external variables. On the other hand, it was observed experimentally that the material’s response is delayed [38]. This delay is more significant for pure metals, but it was also observed for alloys (steels) [39]. These observations led to the development of the models based on the internal variables (IVM). In the IVM, the output is a function of time *t*, with some process parameters **p** and internal variables grouped in the vector **q**: *σ_p_* = f(*t*,**p**,**q**). Since the internal variables represent the state of the material, the IVMs account for the delay of the change in this state due to kinetics of the changes of the microstructure. Dislocation density is the main internal variable. Below is a brief introduction and physical background for modelling deformation, based on the dislocation theory.

### 2.1. Deterministic Model

Introduction of the movement of dislocations as a mechanism for plastic deformation is attributed to Taylor who, in 1934, formulated the expression of the shear stress of the material [40]. His theory has become a basis for all subsequent models. The model presented in (1) originates from the KEM (Kocks–Estrin–Mecking) approach [41,42]. In this approach, the evolution of dislocation populations governs the flow stress and competition of storage and annihilation of dislocations during plastic deformation controls a hardening. After discretization in time, the evolution of the dislocation density $\rho \left(t\right)$ is described by the equation:

(1) $$\rho^{\prime }(t)=A_{1}\dot{\varepsilon }-A_{2}\rho (t){\dot{\varepsilon }}^{1-a_{7}}$$

where: *t*—time, $\dot{\varepsilon }$—strain rate, *A*_1_ = 1/(*bl*), *b*—length of the Burgers vector, *l* = $a_{1}Z^{-a_{2}}$—mean free path for the dislocations, *Z*—Zener-Hollomon parameter, *A*_2_ = *a*_3_ exp[−*a*_4_/(*RT*)], *R*—gas constant, *T*—temperature in K, *a*_4_—activation energy for self-diffusion, *a*_7_—strain rate sensitivity of the dynamic recovery, and *a*_1_, *a*_2_, and *a*_3_—other coefficients.

According to Equation (1), during deformation, the dislocation density increases monotonically until the state of equilibrium between hardening and recovery is reached. The KEM model has been developed during the last few decades with a focus ranging from phenomenological solutions [43] to distinction between various types of dislocations (mobile, trapped) [44]. Several dislocation density reaction models were applied to describe the deformation of various superalloys, such as Ti-alloys [45] and Ni-alloys [46]. The effect of reverse deformation on the evolution of dislocations was investigated in [47]. Several researchers investigated deformation at lower temperatures [48] when recovery is a dominant softening mechanism. 

The theory behind Equation (1) is suitable for modelling processes in which dislocation interactions result in an immediate response of the system. This is true only in the case of the Dynamic Recovery (DRV). In reality, however, an excess of stored energy may lead to Dynamic Recrystallization (DRX) which, in turn, implies a delay in the response of the system. Introducing the DRX follows the one-internal variable model of Sandström and Lagneborg [49]. The required excess of the energy accumulates and can be mathematically simulated by the modification of Equation (1), with a time needed for the development of the dislocation population (stored energy) to a point at which widespread elimination of dislocations is observed. This time is called the critical time for the DRX. The numerical solution of this problem was proposed in [50]. In this model $\rho \left(t\right)$ satisfies the following equation:

(2) $$\rho^{\prime }(t)=A_{1}\dot{\varepsilon }-A_{2}\rho (t){\dot{\varepsilon }}^{1-a_{7}}-A_{3}\rho {\left(t\right)}^{a_{6}}\rho \left(t-t_{cr}\right){\dot{\varepsilon }}^{1-a_{7}}\cdot 1_{\left(t_{cr},+\infty \right)}(t)$$

where: *t_cr_*—time at which recrystallization begins, as a consequence of reaching critical dislocation density *ρ_cr_* and, *A*_3_ = *a*_5_exp[−*a*_6_/(*RT*)], *a*_6_—activation energy for recrystallization, *a*_5_—coefficient, and $1_{\left(t_{cr},+\infty \right)}\left(t\right)$—indicator function of $\left(t_{cr},+\infty \right)$. The indicator function represents the delay in response to the change in processing conditions as it switches on the last part of the equation when *t* > *t_cr_*. Consequently, the Equation (2) is a delayed differential equation (DDE) with respect to time. Besides the numerical solution for this equation, a detailed theoretical analysis of this equation in the case when $\dot{\varepsilon }=1$ was performed in [51]. Classical approaches to the ordinary differential equations (ODE), presented in the literature e.g., [52,53], require some regularity of right-hand side function, usually Lipschitz condition, which is not satisfied by (2). Therefore, Ref. [51] also contains rigorous analysis of the error for the considered equations. It is also interesting that while [54] shows that simple DDEs in the form of (2) cannot be easily used in the modelling of biological or physical phenomena, a natural application to materials science is discussed in [51]. Specifically, it appears that admissible solutions of (2) for the case when $\dot{\varepsilon }=1$ exist are bounded and, furthermore, when $a_{8}\in \left\{0,1\right\},\;$it is possible to derive the exact formulas for $t_{cr}$, which is the time when the recrystallization occurs (Equation (15) in [49]):

(3) $$t_{cr}=\frac{1}{A_{2}}\ln\left(\frac{\rho_{0}-\frac{A_{1}}{A_{2}}}{\rho_{cr}-\frac{A_{1}}{A_{2}}}\right)$$

It is also possible to provide exact mathematical formulas for $\rho \left(t\right)$, the dislocation density (Equations (17) and (18) in [49]):

(4) $$\rho (t)=\exp\left(-A_{2}\left(t-nt_{cr}\right)\right)\cdot \left(\phi_{n-1}(nt_{cr})+\int_{nt_{cr}}^{t}\exp\left(-A_{2}\left(s-nt_{cr}\right)\right)\cdot \left(A_{1}-A_{3}\phi_{n-1}\left(s-t_{cr}\right)\right)ds\right)$$

for $t\in \left[nt_{cr},\left(n+1\right)t_{cr}\right]$ for $a_{8}=0$ and

(5) $$\rho (t)=\exp\left(-\int_{nt_{cr}}^{s}A_{2}+A_{3}\phi_{n-1}\left(s-t_{cr}\right)ds\right)\cdot \left(\phi_{n-1}\left(nt_{cr}\right)+A_{1}\cdot \int_{nt_{cr}}^{t}\exp\left(\int_{nt_{cr}}^{s}A_{2}+A_{3}\phi_{n-1}\left(u-t_{cr}\right)du\right)ds\right)$$

for $t\in \left[nt_{cr},\left(n+1\right)t_{cr}\right]$ for $a_{8}=1$ with the initial value $\rho \left(nt_{cr}\right)=\phi_{n-1}\left(nt_{cr}\right)$ in both cases. Unfortunately, those equations are in the integral form; however, since they exist and are unique, it is justified to use the numerical approximation. Additionally, in [49] authors present a detailed error analysis of the Euler method for DDEs of this kind (Equation (2) satisfies the required conditions, see Lemma 3.1 and Remark 3.7 from [49]). With the auxiliary ODE, the upper bound on the Euler method for a specified horizon can be estimated, as shown by Theorem 3.2 in [49]. The consequence of this analysis is that unique solutions exist for the real-world parameter ranges, which can be efficiently approximated by the Euler method. This ensures correctness of conclusions from numerical simulations.

Beyond the evolution of the internal variables, the solution of the Equation (2) allows calculation of the flow stress *σ_p_* accounting for softening due to recrystallization and recovery. The flow stress is proportional to the square root of the dislocation density:

(6) $$\sigma_{p}(t)=a_{6}bG\sqrt{\rho (t)}$$

where: *a*_6_—coefficient, depending on the material, and *G*—shear modulus.

### 2.2. Case Study—Deterministic Internal Variable Model

As [51] had the rigorous error analysis for the Euler method performed and the stability of this method investigated, it could be confidently applied to a real industrial process. Numerical tests were conducted on a more complicated equation than (2), with strain rate, temperature, and other parameters defined as time dependent. The model coefficients were obtained for DP steel by inverse analysis for the experimental data (hot compression of cylindrical samples performed at constant temperatures and strain rates). The inverse algorithm specified in [37] was also applied. As an example of an application, we considered the industrial process of hot strip rolling because of its heterogeneity of deformation. We decided to use DP steel as a material for the numerical tests. Roll pass data are given in [51]. To calculate distributions of strains, stresses, and temperatures in the roll gap, the authors of [51] used the Thermal–Mechanical Finite Element (FE) model in the macro scale, which is described in detail in [37]. The Levy–Mises flow rule was used as the constitutive law:

(7) $$\sigma =\frac{2}{3}\frac{\sigma_{p}}{{\dot{\varepsilon }}_{i}}\dot{\varepsilon }$$

where: **σ**, $\dot{\varepsilon }$—stress and strain rate tensors, respectively, ${\dot{\varepsilon }}_{i}$—effective strain rate, and *σ_p_*—the flow stress provided by (6). An effect of this approach is briefly presented below.

**Figure 1:**
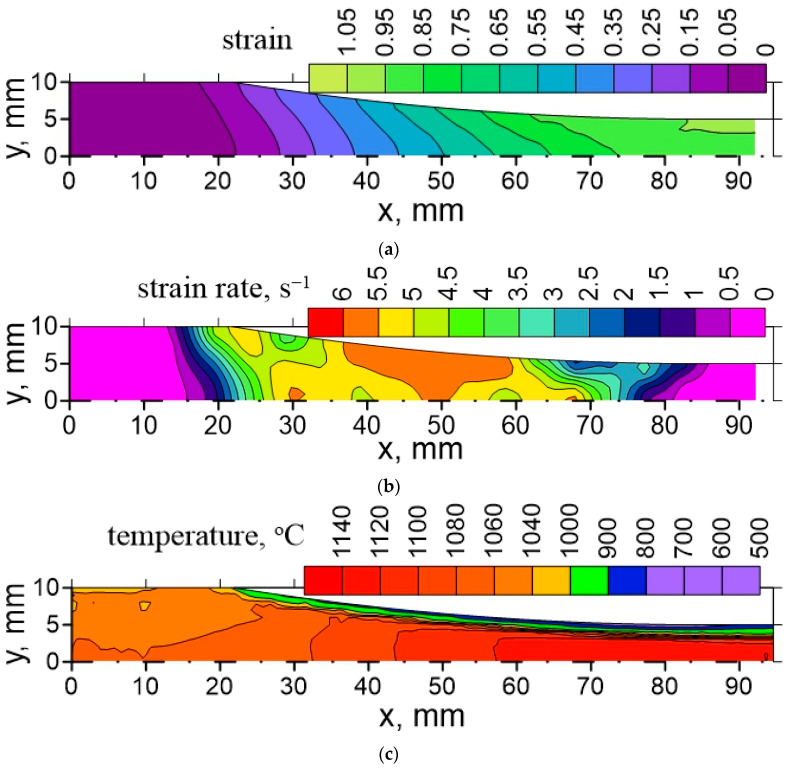
Calculated distribution of strain (**a**), strain rate (**b**) and temperature (**c**) in roll gap for rolling of DP steel [51].

**Figure 2:**
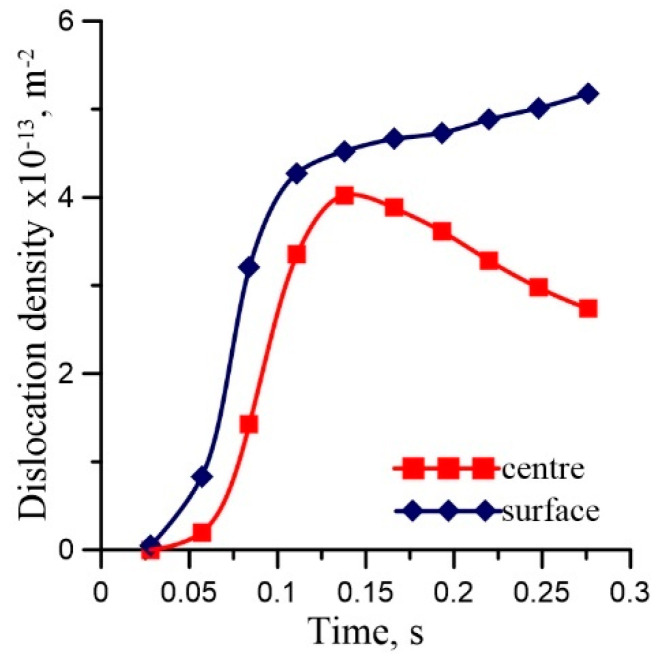
Calculated variations of dislocation density along the two flow lines in roll gap during rolling of DP steel.

Distributions of the strain, the strain rate and the temperature in the roll gap, calculated by the FE code, are shown in Figure 1. Since the process is symmetrical with respect to the horizontal axis, only a top part of the roll gap is shown. Equation (2) was solved along the flow lines in the roll gap using current local values of the strain rate and the temperature calculated by the FE model. The results for the two lines—one located in the center of the strip and the other 2 mm below the surface—are presented in Figure 2. Starting dislocation density *ρ*_0_ equals 10^4^ m^−2^. The entry temperature is equal to 1060 °C. The results were determined using the Euler method with time-dependent coefficients $A_{1}\left(t\right),A_{2}\left(t\right),A_{3}\left(t\right)$. Analysis of these results shows that they correctly reproduce the effect of distinct temperature and strain rate histories for the two considered areas. One can see in Figure 2 that, in the center of the strip, while the temperature increases (Figure 1) due to deformation heating, the dislocation density decreases as an effect of the dynamic recrystallization. Additionally, the dislocation density in the surface increases during the temperature decrease because of heat transfer to the cool roll. Consequently, the critical dislocation density for DRX is higher and reached later. In the central part, the strain rate decreases monotonically by cause of the monotonic deformation of this part. The results from the Euler method presented in Figure 2 replicate proper material behavior in these conditions of the deformation. For a detailed description, see [51].

## 3. Stochastic Model for Hot Forming

The delayed differential Equation (2) considered in the previous section is built on critical time *t_cr_*, which is an artificial parameter. The reason for this situation is that in a real material, the onset of the recrystallization may occur in a different time in various parts (various material points) and this process is highly stochastic in nature. The model based on Equation (2) describes the process on average; therefore, it cannot completely and adequately reproduce the stochastic behavior of the material, providing only partial insight into the process. Beyond this, the critical time *t_cr_* is not a physical quantity. It is important to realize that using this time in the model only allows use to average the material response to deformation, which is a weak point of the model (2). A stochastic approach based on Equation (2) is a method to avoid the artificial critical time and build the model, which is closer to the reality.

In common understanding, the recrystallization is a process which is responsible for replacement of a deformed microstructure with high dislocation density by new grains practically free of dislocations. In general, the recrystallization is considered a specific type of transformation. Processes of phase changes in metallic materials are composed of nucleation and growth stages. It means that the two phases can coexist during the recrystallization. In the recrystallization, the deformed part of the material with increased dislocation density is considered an old phase and the part of the material with rebuilt microstructure and free of dislocations is regarded a new phase. Two types of recrystallizations are distinguished. The first is dynamic recrystallization (DRX), which occurs during the deformation. The second is static recrystallization (SRX), which occurs after the deformation. In many industrial processes, recrystallization is considered a phenomenon which, to a large extent, determines the final microstructure and mechanical properties of the alloys.

### 3.1. State-of-the-Art in Stochastic Approach to Modelling Recrystallization

The research on recrystallization dates back to the 19th century; the physical basis of the theory of the recrystallization was summarized in [55]. During hot deformation of metals and alloys, a competition of storage (strain hardening) and annihilation (recovery) of dislocations leads either to saturation (for high SFE—Stacking Fault Energy) or tends to increase the energy stored in the material (for low SFE). When the energy stored in the microstructure is high enough, new grains nucleate. In parallel, migration of grain boundaries occurs as a result of capillary effects and stored energy gradients across interfaces [56]. Combining those mechanisms leads to the so-called Dynamic Recrystallization (DRX) [55]. The tendency toward DRX increases with decreasing Zener–Hollomon parameter. Recrystallization can also occur at relatively lower temperatures after deformation (SRX).

Different deterministic mean-field phenomenological models of recrystallization, based mainly on the JMAK (Johnson–Mehl–Avrami–Kolmogorov) equation, were proposed during the latter half of the 20th century. The predictive capabilities of these models are limited and they are constrained to average values of microstructural features. The development of numerical methods led to more sophisticated models that explicitly reproduce microstructural evolutions. These are the so-called full-field models based on phase-field or Cellular Automata and LSM approaches. The full-field models accurately describe recrystallization; however, a high computational cost is their major limitation. In contrast, the main advantage of all mean-field models is their very low computational cost.

The problem of the stochastic character of the nucleation during recrystallization has been studied by researchers [57]; a majority of the published solutions are dedicated to the full-field models, in which microstructure is represented explicitly by the Cellular Automata space [58] or by the Representative Volume element (RVE) [59], with the Level Set Method applied to describe the motion of the interface [60]. Extended crystal plasticity was used in [57]. Several researchers attempted to capture the stochastic nature of recrystallization by adopting an MCP-like approach (Monte Carlo–Potts) [61]. A comprehensive review of these models can be found in the PhD thesis [62]. As has been mentioned, the full-field models require long computing times and their application to the optimization of processes is inefficient. Stochastic mean-field models can be proposed as a compromise between deterministic phenomenological laws and full-field models.

As far as stochastic mean-field recrystallization models are considered, the stochastic vertex model of the recrystallization is proposed in [63]. In this model, the structure of grains is defined by a set of vertices (triple points). It is assumed that a grain boundary can move during recrystallization only if the stored energy difference between two neighboring grains is greater than some critical value. The deterministic equations of triple point movements are replaced by the Monte Carlo model. The model of [63] was applied to predict the recrystallization texture of copper.

Few mean-field solutions are based on an implicit description of the microstructure by a set of representative grains defined by their size and dislocation density [64]. Each grain is considered in a Homogeneous Equivalent Medium (HEM) and evolution is governed by its interaction with the HEM. This model was used to describe recrystallized fractions and grain size distributions, though the agreement with the measurements is poor. This poor agreement is due to the fact that all grains which have nucleated at a given time have the same size and dislocation density. In contrast, in a real microstructure, each grain develops depending on its neighborhood. To eliminate this drawback, in [25] a topological approach for the mean-field stochastic modelling of DRX was proposed. The authors named their model NHM (NeighborHood Model). The NHM is an upgrade of the model proposed in [64] based on the consideration of a particular neighborhood for each grain instead of the whole average microstructure as HEM. Presented simulations confirmed the low computing cost of the HEM, which enables the simulation of the microstructural evolutions in less time than full-field models.

### 3.2. Mathematical Background

A model that incorporates the stochastic character of the phenomena, thanks to the application of the Monte Carlo method, may predict probability distribution of parameters (dislocation density, grain size). This is its main difference to the models without stochastic characters, which were only able to predict parameters’ averages. Nevertheless, when using it we need to determine the number of Monte Carlo realizations in order to properly recover properties of real process in the histogram. Before this action, we need to establish a method for measuring the performance of the model.

As has been mentioned in the introduction, minimizations of the computation cost is one of crucial concerns in modelling. However, in this model it should be taken with extreme caution. Due to a limited number of solutions, a reliable histogram could not be created. That problem stems from the possibly huge impact of the random factor. Moreover, the results are not reproducible. Thus, to find the optimal number of solutions and bins, we decided to use Mean Average Percentage Error and its slightly modified version, Weighted Mean Average Percentage Error, to describe distance between histograms, giving us information about the differences between the considered bins. Moreover, to be more precise, we used Earth Mover’s Distance ranking function, which is more sensitive in detecting shape differences in the compared histograms. A detailed description of this approach can be found in [35].

The big-picture overview follows several steps. Firstly, we simulated the process using the model and, as a result, obtained distribution through a histogram. We then collected analogous data from the experiments and represented them on a histogram. The solution depended on a formulation of the objective function measuring the difference between the two histograms [35] for a given parameter, namely those delivered by the model with the coefficients returned by optimization and obtained from experimental measurements. An arbitrary ranking metric d for measuring the distance between distributions (histograms), e.g., Bhattacharyya distance $d_{H}$ [35,65], might be used in this context. Consequently, the objective function defined in this way can be associated with a measure of the optimization quality for the stochastic problem. Importantly, the definition of the objective function chosen has to be adequate for the considered problem.

### 3.3. Main Equations of the Model

As we earlier described, a desired extension of the Equation (2) is introducing stochastic variables and combining phenomenological microstructure evolution equations with statistics accounting for the random character of the recrystallization. The stochastic approach is based on substituting the critical time *t_cr_* with a nucleation probability. To complete this task, authors of [51] first presented the Equation (2) in a finite difference form:

(8) $$\rho \left(t_{i}\right)=\rho \left(t_{i-1}\right)+\left(A_{1}\dot{\varepsilon }-A_{2}\rho \left(t_{i-1}\right){\dot{\varepsilon }}^{1-a_{7}}-A_{3}\rho {\left(t_{i-1}\right)}^{a_{6}}\rho \left(t_{i-1}-t_{cr}\right)\cdot 1_{\left(t_{cr},+\infty \right)}\left(t_{i-1}\right)\right)\Delta t$$

where: *t_i_*—the time of *i*th iteration and Δ*t*—a time step.

The artificial delay term used in the model (8) is now replaced by a more realistic stochastic variable *ξ*(*t_i_*). This way, the evolution of the dislocation density is no longer a deterministic function of time, becoming stochastic:

(9) $$\rho \left(t_{i}\right)=\rho \left(t_{0}\right)\left(1-\xi \left(t_{i}\right)\right)+\left(\rho \left(t_{i-1}\right)+\left(A_{1}\dot{\varepsilon }-A_{2}\rho \left(t_{i-1}\right){\dot{\varepsilon }}^{1-a_{7}}\right)\Delta t\right)\xi \left(t_{i}\right)$$

This new model contains analogous elements, as shown in deterministic model (1). In particular, we still use coefficients *A*_1_ and *A*_2_ which, similarly to (1), represent hardening and recovery in the material (see [51] for more details). At the same time, we removed coefficient *A*_3_ connected with critical time in (2); its role is taken by *ξ*(*t_i_*). Its distribution is defined by the following formulas:

(10) $$\begin{array}{l} P\left(\xi \left(t_{i}\right)=0\right)=\left\{\begin{array}{ll} p\left(t_{i}\right) & \mathrm{if}\;p\left(t_{i}\right)<1\\ 1 & \mathrm{otherwise} \end{array}\right.\\ P\left(\xi \left(t_{i}\right)=1\right)=1-P\left(\xi \left(t_{i}\right)=0\right) \end{array}$$

In Equation (10), the function *p* depends on the present state of the material at a given time step, which strongly affects the probability of recrystallization of a material. This function was defined based on the expert knowledge about nucleation probability during recrystallization [57]. To avoid long computing times, the formula for probability was limited only to statistical evaluation of selected phenomena (stored energy, grain boundaries) by neglecting others (crystallographic orientation). In the first approach, the probability of the nucleation was based on the simple homogeneous Poisson Point Process, leading to the following equation:

(11) $$p=a_{4}\rho^{a_{6}}\frac{3\gamma \tau }{D}\exp\left(\frac{-a_{5}}{RT}\right)\Delta t$$

In Equation (11), coefficient *γ* represents the migrating boundary area related to the grain size. The time dependence of this fraction is discussed in [49]. It is the effect of two opposing phenomena. The first is an increase in the fraction of the grain boundary area which is migrating (1 − exp(*X*(*t_i_*)). The second is the impingement factor (1 − *X*(*t_i_*)), which takes into account the fact that the migrating boundary encounters ever-more regions which have already been recrystallized. Since in the Poissonian model of the nuclea-tion the recrystallized volume fraction *X*(*t_i_*) is not known for individual Monte Carlo points, it was substituted by the probability **P**(*ξ*(*t_i_*_−1_) = 0). Thus, in the first approach the migrating boundary area related to the grain size is expressed by the following equation:

(12) $$\gamma \left(t_{i}\right)=\left(1-\exp{\left(-P\left(\xi \left(t_{i-1}\right)=0\right)-q\right)}^{a_{8}}\right)\left(1-P\left(\xi \left(t_{i-1}\right)=0\right)\right)$$

where: *q*—a small artificial number to avoid zero value of *γ* in the case **P**(*ξ*(*t_−_*_1_) = 0) = 0. The authors of [57] set it to *q* = 0.1 and also define *ξ*(*t*_0_) = 0.

The Equation (9) changed deterministic values of *ρ* into stochastic variables with some probability distribution in each step. Since we do not have an analytic formula, an alternative approach to reveal these distributions is conducting Monte Carlo simulations. Namely, we compute a large number of particular trajectories of (9), depending on randomly generated values of *ξ*(*t_i_*) according to rules in (13). Initial values *ρ*(*t*_0_) are drawn from the Gauss distribution, with the expected value selected as 10^4^ m^−2^ for start time *t*_0_ = 0. The results of these computations were then aggregated into histograms, one for each time step *t_i_*.

In the second approach, the nucleation probability was based on the non-homogeneous Poisson Point Process, assuming that the growing recrystallized grains cause a decrease in the nucleation of new grains. The following equation was proposed as a substitution of the Equation (12):

(13) $$\gamma \left(t_{i}\right)=\left(1-\exp{\left(-X\left(t_{i-1}\right)-q\right)}^{a_{8}}\right)\left(1-X\left(t_{i-1}\right)\right)$$

where: *X*(*t_i_*_−1_) = *n_X_*/*n_MC_*, *n_X_*—number of points, which have recrystallized before the time *t_i_*, and *n_MC_*—number of Monte Carlo points.

The model contains 16 coefficients, which are grouped in the vector **a**. The coefficients were identified using an inverse solution for the experimental data, some of which were in the form of histograms describing the grain size.

### 3.4. Identification

A standard method in modelling is the so-called inverse problem [66,67], where we try to identify coefficients of the model. The model we introduced earlier depends on the coefficients **a** = {*a*_1_, …, *a*_16_}. The general algorithm of identification is well known [37] and beyond this survey. It is sufficient to say that we have to perform an optimization task, with vector **a** becoming input variable of objective function [31]. The authors in [35] proposed this function to be:

(14) $$\Phi \left(a\right)=d\left(H_{c}\left(a\right),H_{m}\right)$$

where: *H*_c_(**a**)—histogram calculated on the basis of several model simulations of, *H_m_*—histogram measured in the experiment, and *d*—a ranking function comparing two histograms.

For practical reasons, the approach in [35] relied on measurements of forces in the compression tests which, using inverse algorithm [67], were transformed to the flow stress (*σ_m_*). As its counterpart, model flow stress (*σ_c_*) was obtained by the application of (6). This way, an extended version of (15) was proposed as an objective function:

(15) $$\begin{array}{l} \Phi (a)=\Phi_{\sigma }(a)+\Phi_{D}(a)\\ \Phi_{\sigma }(a)=w_{\sigma }\sum_{i=1}^{Nt}d\left(\sigma_{ci}\left(a\right),\sigma_{mi}\right)\\ \Phi_{D}(a)=w_{D}\sum_{i=1}^{Nt}d_{H}\left(H_{ci}\left(a\right),H_{mi}\right) \end{array}$$

where: *Nt*—number of tests and *w_σ_*, *w_D_*—weighted coefficients.

In Equation (16), the distance *d*(*σ_ci_*(**a**), *σ_mi_*) between measured and calculated average dislocation density in the *i*-th experiment was measured as the mean square root error (MSRE):

(16) $$d\left(\sigma_{ci}\left(a\right),\sigma_{mi}\right)=\frac{1}{Ns}\sqrt{\sum_{j=1}^{Ns}{\left(\frac{\sigma_{cij}(a)-\sigma_{mij}}{\sigma_{mij}}\right)}^{2}}$$

where: *Ns*—number of sampling points for measurements of the loads in the *i*-th test.

The Bhattacharyya metrics [65] were used to calculate the distance between two histograms *d*(*H_ci_*(**a**),*H_m_*). We refer the reader to [31] for more details on the Inverse Analysis (IA) for the stochastic model and the Sensitivity Analysis (SA), which preceded inverse analysis.

### 3.5. Numerical Tests

The numerical tests performed in [35] and the main conclusions following from these tests are described below. The objective of the tests was to select the optimal numerical parameters of the solution and evaluate the reliability of the inverse solution for the objective function based on the histograms. Simulations for various numbers of bins in the histograms (*n_b_*), Monte Carlo points (*n_MC_*), and time steps (*n_t_*) were performed and the following optimal values were selected: *n_b_* = 10 and *n_t_* = 20,000. In order to test the impact of a time step on a solution, the cumulative probability of dislocation density reduction after the given time was computed in [35]. Roughly speaking, it is the probability that *ξ*(*t_i_*) (Equation (10)) obtained the value *ξ*(*t_i_*) = 1 at least once during the process. While probabilities in Equation (10) depend on length of time step, the results of the numerical tests in [35] showed cumulative probabilities that looked similar despite different time steps. The optimal number of time steps was obtained by adapting these steps to the temperature variations so that the time step does not exceed 0.1 s and the temperature change in the one-time step does not exceed 0.1 °C. The optimal parameters were selected by searching for the balance between the accuracy (repeatability of distributions, convergence) and the computing time. Five various metrics were analyzed in [35] as a measure of the distance between histograms and, as a result, the Bhattacharyya function [65] was selected as the best performing. Its utility was independent of the optimization method (two were tested: Particle Swarm Optimization (PSO) and Nelder–Mead Simplex Method).

## 4. Case Studies—Stochastic Model

The stochastic model we described earlier will be now applied to a simulation of the two hot forming processes. The first is a laboratory compression test with varying strain rates. The second is the industrial hot strip rolling process.

### 4.1. Varying Strain Rate Test

**Figure 3:**
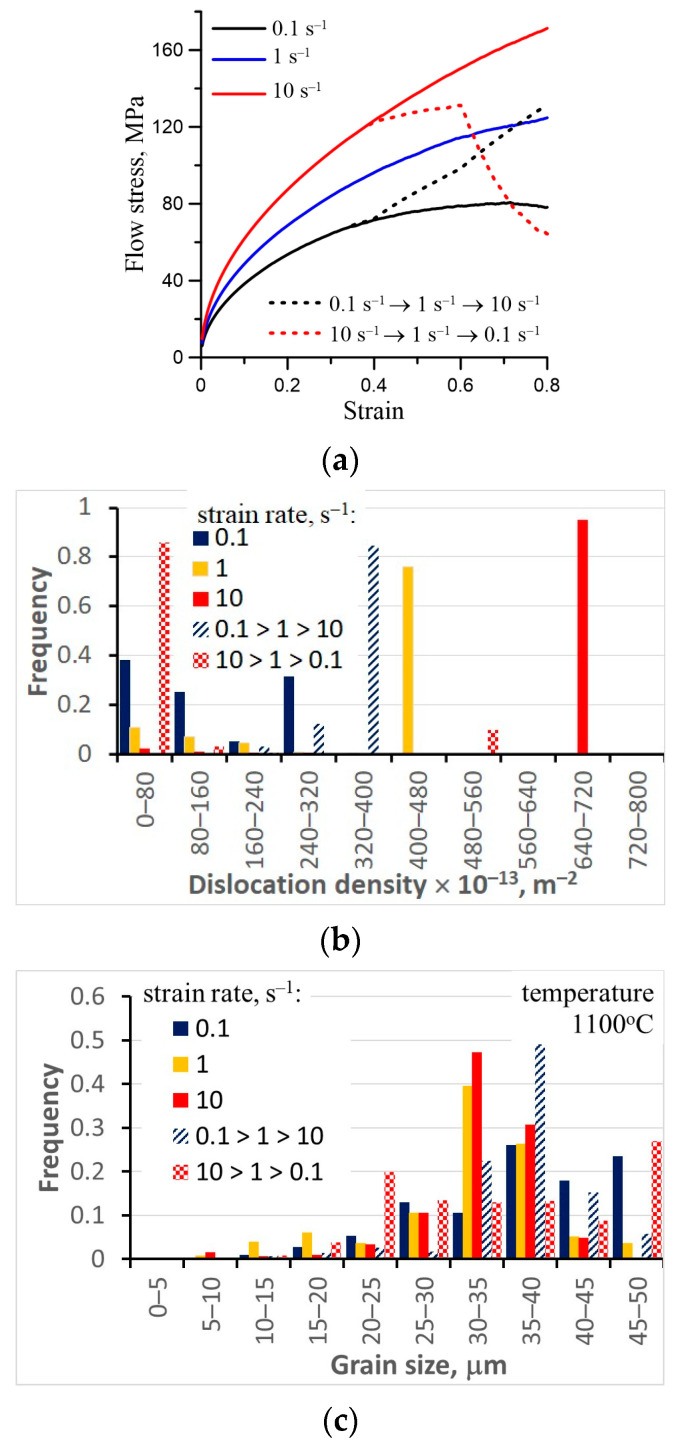

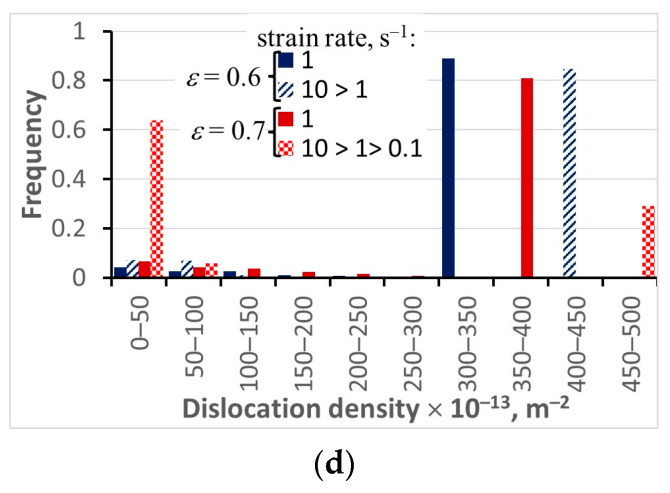
Changes of flow stress during various tests (**a**), histograms of dislocation density (**b**), and grain size (**c**) during constant and varying strain rate tests. Changes of dislocation density directly after change in strain rate (**d**).

In this test, the strain rate was changed by order of magnitude at various stages of the deformation. The objective of the simulations of this test was to show the model’s capability to account for the history of the deformation and predict a delay in the material’s response, which has been observed experimentally by many researchers [38,39]. Compression of the sample measuring ϕ10 × 12 mm was considered. The material was carbon steel S355J2 (see [68] for the coefficients of the model for this steel). Below we present the results for the tests when the strain rate is changed by an order of magnitude twice during the total strain of 0.8. The first change between 0.1 s^−1^ and 1 s^−1^ was at the strain of 0.4 and the second change between 1 s^−1^ and 10 s^−1^ was at the strain of 0.6. Symmetrically, simulations were performed for the strain rate decreasing from 10 s^−1^ to 1 s^−1^ and to 0.1 s^−1^. The average austenite grain size after pre-heating was 35 μm and the sample temperature was 1150 °C, which meant that, in the slow test at the strain of 0.4, the dynamic recrystallization (DRX) had already begun. The Finite Element (FE) program was applied to calculate current local temperatures and strain rates in the sample, accounting for the inhomogeneity of deformation. Our in-house FE program [69] was used and it was possible to solve the stochastic model in each Gauss integration point in the FE mesh using calculated temperatures and strain rates as inputs. The results for the center of the sample are presented below. Changes in the flow stress in the varying strain rate tests are shown in Figure 3. A delay in the material response is well seen in this figure. Histograms of the dislocation density after these tests are shown in Figure 3b and histograms of the grain size are in Figure 3c. The histograms’ full bars represent dislocation density and grain size after the constant strain rate tests. The bars with a pattern represent dislocation density and the grain size after the varying strain rate tests.

It is seen in Figure 3b that DRX is launched during the slow test ($\dot{\varepsilon }$ = 0.1 s^−1^). The recrystallized volume fraction is above 60%. However, after the strain of 0.4 the strain rate is increased and the effect of the DRX is negligible. In contrast, when the strain is decreased during the test to 0.1 s^−1^, the flow stress (dislocation density) decreases below the value predicted for the constant strain rate of 0.1 s^−1^ test. This outcome is due to the fact that, during the fast part of the test, energy is accumulated in the material (dislocation density increases). This energy accelerates the DRX during the final slow part of the test. This observation is confirmed by Figure 3d, where changes of the dislocation density right after the strain rate change are shown.

As far as the grain size is considered, an increase in the strain rate causes a decrease in the grain size, which could be expected. The largest grain size was obtained for the tests in which the final part of the deformation was with the strain rate of 0.1 s^−1^.

The general conclusion from these simulations is that after the change in the strain rate, the state of the system had not reached the state which was predicted for the constant strain rate deformation.

### 4.2. Industrial Hot Strip Rolling Process

The hot strip mill composed of a reverse roughing mill, 6-stand continuous finishing mill and 2-section laminar cooling was considered [70]. Rolling of the strip 1500 × 3 mm was simulated. The material was steel DP600 with the symbol S406 in publication [71]. The work roll radius was 400 mm in all stands and the distance between stands was 5 m. The rolling schedule for the finishing mill was: 67 → 40.6 → 19.1 → 9.4→ 5.4 → 3.7 → 3 mm.

**Figure 4:**
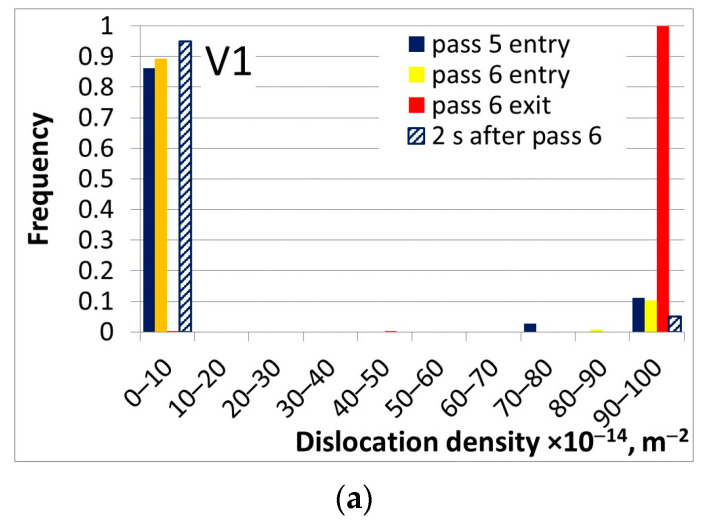

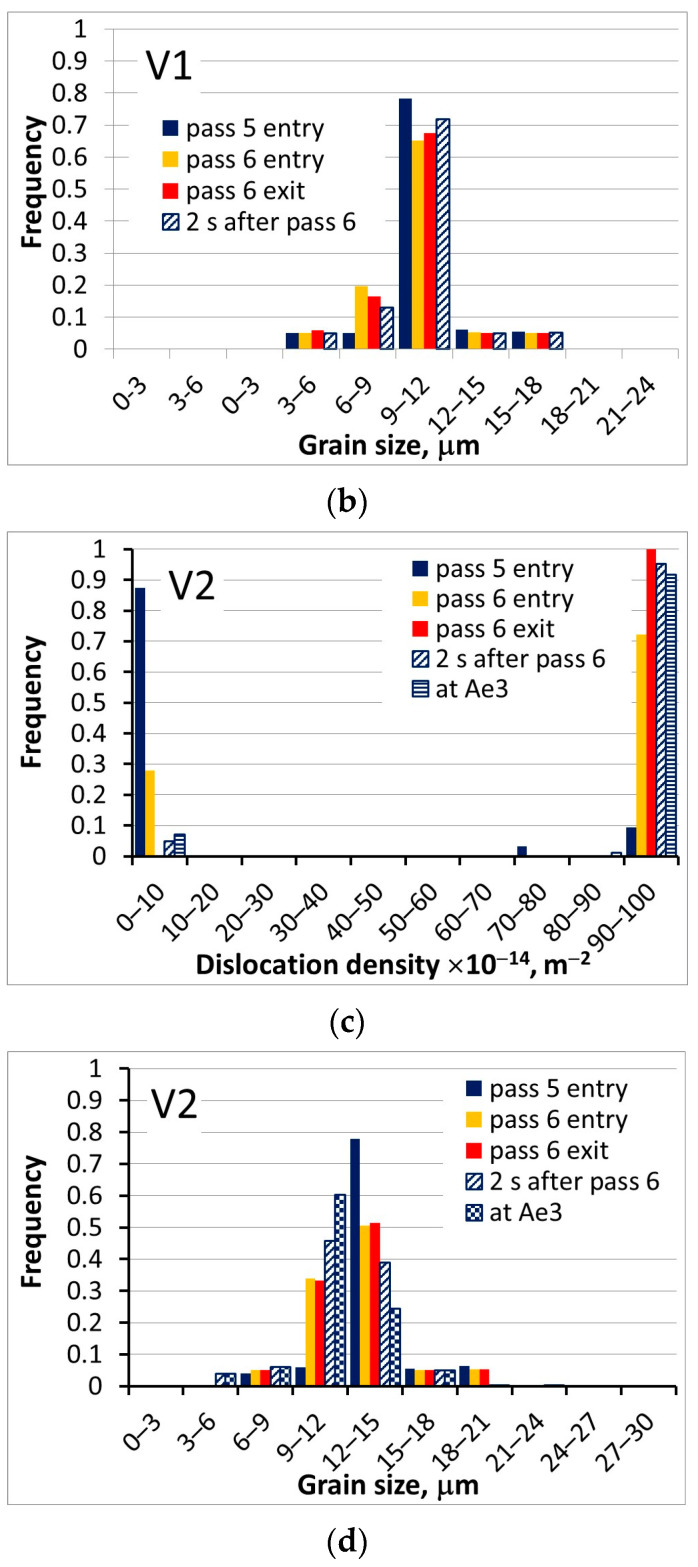
Calculated histograms of dislocation density (**a**,**c**) and austenite grain size (**b**,**d**) in final two passes of hot strip rolling according to rolling schedule V1 (**a**,**b**) and V2 (**c**,**d**).

Two rolling strategies were considered: (i) classical rolling with the end of rolling temperature about 900 °C (V1); and (ii) rolling with an ultra-fast cooling system between stands 4 and 5 and stands 5 and 6. The temperature at the end of rolling was below 850 °C (V2). In both cases, phase transformations during the following laminar cooling were simulated. Calculated time-temperature profiles and load parameters for both variants are shown in [70]. An small effect of additional cooling between stands on loads was evaluated in that publication. The selected results obtained from the stochastic model for the finl two passes of the finishing mill are shown in Figure 4. These results allow us to compare different rolling strategies as far as microstructure evolution is considered. It is seen that in the schedule V1, recrystallization is completed during all interpass times and almost the whole material has dislocation density in the lowest bin. The results in Figure 4c indicate that an additional cooling leads to a partial recrystallization between stands 4 and 5 and a lack of recrystallization after the last stand. A decrease in the end of rolling temperature causes an increase in the dislocation density at the beginning of transformations (temperature *A_e_*_3_).

Comparison of the grain size for the two rolling strategies shows that a decrease in the temperature in stands 5 and 6 leads to a decrease in the austenite grain size after rolling. It can be concluded that the model can be used to predict histograms of the micro-structural features as a function of the process parameters. The microstructural features predicted by the model have a direct influence on the phase transformations during cooling.

## 5. Future Applications of the Stochastic Model

The control of phase transformations determines the properties of hot-rolled products during cooling. In order to account for the effect of the heterogeneity of the microstructure on the properties, the stochastic model of the phase transformation is needed. A model which considers the stochastic character of nucleation is the objective of our current research. In the meantime, we performed simulations of phase transformations using a deterministic model with the stochastic input in the form of histograms of the dislocation density and the grain size as an input. Simulations of the laminar colling of strips and cooling of rods in the Stelmor system were performed; the results are described in the following two sections.

### 5.1. Laminar Cooling of Hot Rolled Strips

**Figure 5:**
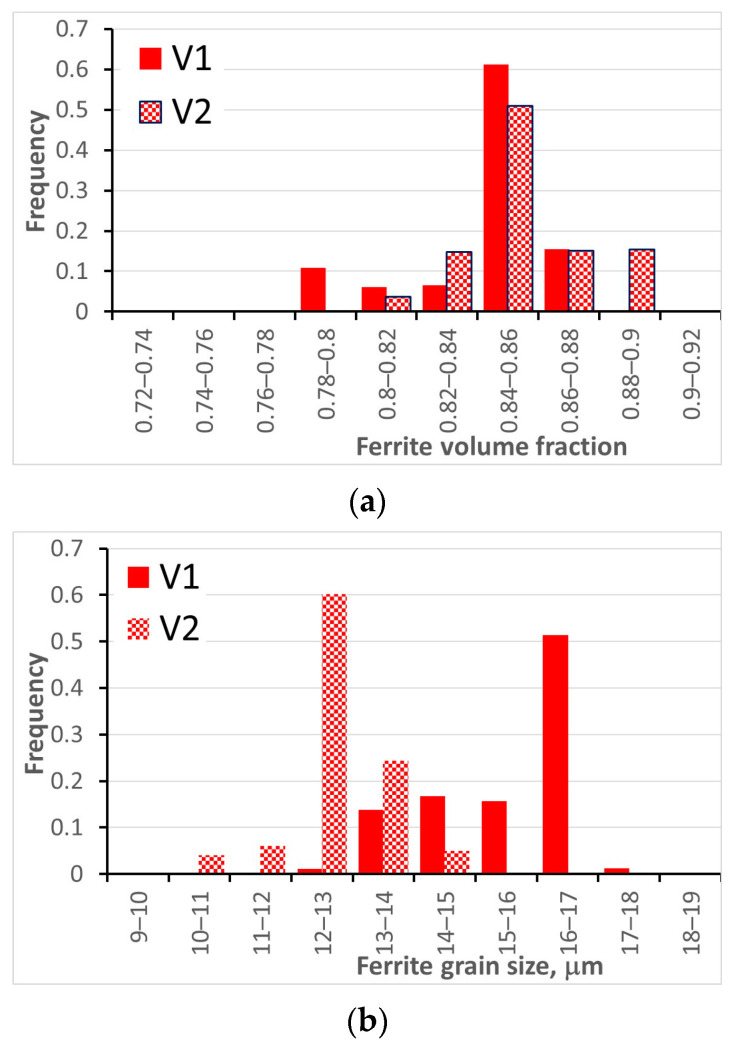
Calculated histograms of ferrite volume fraction (**a**) and ferrite grain size (**b**) after laminar cooling of steel strip.

Phase transformations were simulated during laminar cooling using a deterministic model with stochastic input (histograms calculated by the stochastic model for hot deformation). The deterministic model of phase transformations in the steel DP600 is described in [71]. The typical laminar cooling system composed of two sections and described in [72] was considered. This system allows a three-stage cooling sequence: fast/slow/fast cooling. In consequence, the DP microstructure composed of ferrite and martensite can be obtained. Since the input data for the deterministic phase transformation model were stochastic, the calculated phase composition was obtained in the form of histograms, which are shown in Figure 5a. It is seen in this figure that the deformation of the austenite (V2) results in an increase in the ferrite volume fraction. Beyond the ferrite, martensite and a small amount of bainite (below 0.02) were predicted by the model. The ferrite grain size was also calculated by the model. The deterministic equation proposed in [73], which accounts for the effect of the austenite deformation, was used. Since the input parameters (grain size, dislocation density) are stochastic; similarly to the previously presented experiments, the ferrite volume fractions and the ferrite grain size were obtained in the form of histograms. As expected, deformation of the austenite accelerates nucleation of the ferrite and the ferritic transformation is faster. As a consequence, volume fraction of ferrite is larger for the rolling schedule V2 (Figure 5a). Moreover, the distribution of the ferrite volume fraction can be considered as an uncertainty of the predictions due to uncertainty in the microstructural parameters after hot forming. Analysis of the results in Figure 5b shows that finer ferrite grain size was predicted for the rolling schedule V2.

### 5.2. Cooling of Rods in the Stelmor System

**Figure 6:**
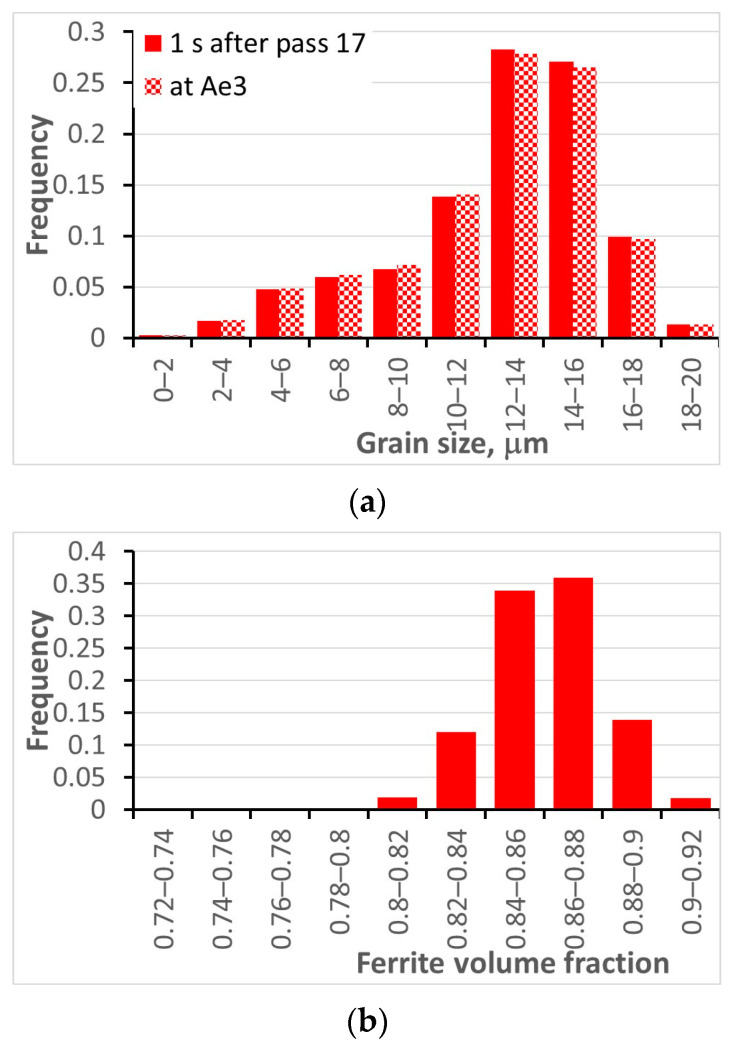
Calculated histograms of ferrite volume fraction (**a**) and ferrite grain size (**b**) after cooling of steel flat rods.

Simulation of phase transformations during cooling in the Stelmor system was performed. As it has been outlined in the previous section for the hot strip rolling, a deterministic phase transformation model with stochastic input was used. Simulations of the last few passes during the hot rolling of rods were performed by the Thermal–Mechanical FE program coupled with the stochastic model of the microstructure evolution; histograms of the dislocation density and the grain size after rolling were also calculated. Details of this process are described in [74] and are not repeated here. Steel S355J2 was the investigated material. Since the recrystallization was completed after the last stand, only the effect of the grain size on the transformations kinetics was considered. The Stelmor system described in [74] was considered and the accelerated cooling cycle W440 shown in Figure 4 in that publication was simulated. The histograms of the austenite grain size after rolling are shown in Figure 6a. It is seen that the grain size has reasonably wide range of variance, which is due to non-uniform rolling conditions and heterogeneity of the microstructure after rolling. The histogram of the ferrite volume fraction is shown in Figure 6b. The remaining material was pearlite. The histogram in Figure 6b can be considered as an uncertainty in the prediction of the phase composition due to heterogeneity of the microstructure after hot rolling.

## 6. Summary

The main objective of this survey paper was a review of the stochastic modelling of the microstructure during hot strip rolling that is used in contemporary research. As an example of applications, we presented a stochastic model for the hot deformation. When this model is coupled with the FE program, it can be applied to any hot forming process. The phase transformations part is still developed in present research, providing a ground for further advancements. Considered approach defines a deterministic phase transformation model with stochastic input data (histograms of the dislocation density and the grain size calculated by the hot deformation model). Let us briefly summarize main aspects of presented models and applications:

- Capability to predict histograms of different microstructural features instead of their average values is the main advantage of the model. It reflects real-world situations more adequately than models using averaged values of dislocation density or grain size.
- All considered models are classified as mean-field models that do not needing explicit representation of the microstructure. In consequence, their computing costs are low.

The numerical tests of the model allowed us to select optimal numerical parameters, which give a balance between accuracy and computing costs. The following observations were made:

- Due to the stochastic nature of Equation (9), the repeatability of histograms depends on the number of points. However, it can be kept at a reasonably low level depending on the design of the experiment. In considered cases, we observed that 20,000 simulations with 10 bins allowed us to reduce differences between generated histograms to the level of 3%.
- In the Inverse Analysis, error on target to computed histogram was decreased to 6%, which is a reasonable score bearing in mind that comparison of the two histograms at exactly the same parameters can result in 3% difference.

The presented models behave well on the varying strain rate test and results of validation are satisfactory. Predictions of the models were in qualitative agreement with published information about these tests, which ensures the utility of the considered models in practical applications in material science. In particular, presented models can be used to simulate industrial hot strip rolling. The results are in agreement with our knowledge about this process, confirming the model’s capability to support a design for the optimal rolling technology.

Accounting for the random character of the nucleation during phase transformation is still an open problem for further research. When successful, it can lead to a more accurate description of metallurgical processes, keeping computing times low.

## Data Availability

The data that support the findings of this study are contained within the article.
